# Influence of Cilioretinal Arteries on Flow Density in Glaucoma Patients Measured Using Optical Coherence Tomography Angiography

**DOI:** 10.3390/jcm12072458

**Published:** 2023-03-23

**Authors:** Julian Alexander Zimmermann, Jens Julian Storp, Raphael Diener, Moritz Fabian Danzer, Eliane Luisa Esser, Nicole Eter, Viktoria Constanze Brücher

**Affiliations:** 1 Department of Ophthalmology, University of Muenster Medical Center, 48149 Muenster, Germany; 2 Institute of Biostatistics and Clinical Research, University of Muenster, 48149 Muenster, Germany

**Keywords:** optical coherence tomography angiography, glaucoma, cilioretinal artery, severity, flow density, vessel density

## Abstract

It has long been speculated whether the presence of a cilioretinal artery (CRA) can influence the development of glaucomatous damage in patients with open-angle glaucoma. Studies involving healthy patients have shown a change in flow density (FD) depending on the presence of a CRA. Similarly, studies that compared the optical coherence tomography angiography (OCTA) results of healthy controls and glaucoma cohorts identified a reduction in FD in certain retinal layers for glaucoma patients. These observations raise the question of whether FD is altered in glaucoma patients depending on the presence of CRA, with possible implications for the progression of glaucomatous damage. In this prospective study, 201 eyes of 134 primary and secondary open-angle glaucoma patients who visited the Department of Ophthalmology at the University of Muenster Medical Center, Germany were included. The patients were allocated to different groups according to the presence of CRAs and the level of glaucoma severity. The FD results obtained using OCTA for the CRA and non-CRA groups were compared. While FD differed noticeably between the CRA and non-CRA cohorts in the deep macular plexus, no differences in FD were observed between the two groups when adjusted for glaucoma severity. In both the CRA and non-CRA eyes, increasing glaucoma severity correlated most strongly with a reduction in peripapillary FD. Our results suggest that the presence of CRAs does not significantly affect retinal perfusion in glaucoma patients.

## 1. Introduction

Glaucoma is a leading cause of irreversible visual impairment worldwide. This disease is associated with a loss of retinal ganglion cells and subsequent characteristic peripheral vision loss [1]. Intraocular pressure (IOP) is considered as the main risk factor involved in the pathogenesis of glaucoma, causing mechanical stress to the nerve fiber bundle. However, the “mechanical theory” does not apply to all cases of glaucoma (e.g., normal-tension glaucoma). The “vascular theory” identifies changes in ocular perfusion as the cause of the degeneration of the optic disc [2,3].

**Figure 1:**
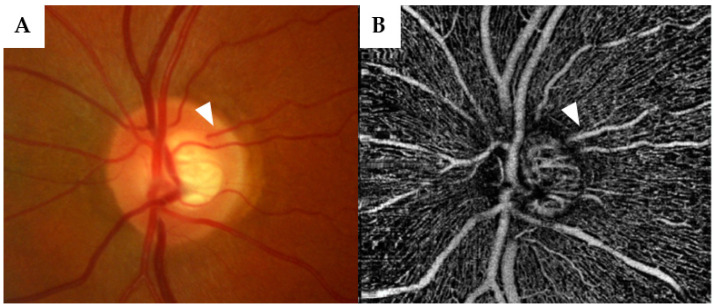
Fundus imaging (**A**) and optical coherence tomography angiography (OCTA) (**B**) in a 51-year-old male primary open-angle glaucoma patient show a cilioretinal artery (arrowhead) at the temporal rim of the optic nerve head without a connection with the central retinal artery.

The central retinal artery is responsible for the arterial blood supply to the inner layers of the retina, while the outer layers of the retina are supplied blood via the choroidal vessels. First described by Müller in 1856, cilioretinal arteries (CRAs) (Figure 1)—an anatomical variant—originate from the short posterior ciliary arterial circulation, or in some cases, directly from the choroid [4,5]. In the majority of cases, a cilioretinal vessel supplies blood to parts of the macula. 

Optical coherence tomography angiography (OCTA) is a novel imaging technology that facilitates the quantification of the retinal vasculature by measuring flow density (FD) [6,7]. OCTA trials have identified several glaucoma-associated alterations in retinal FD [8,9]. The majority describe a reduction in macular and peripapillary FD results in comparison with healthy individuals [10,11,12,13]. Furthermore, these changes have been reported to be severity-dependent [14,15]. Later studies have identified OCT angiographic changes in glaucoma patients to correlate with structural OCT changes and visual field loss [16,17].

Recent trials assessing the eyes of healthy individuals have described macular and peripapillary FD to be higher in the presence of CRAs compared to control eyes without the vessel [18,19].

This raises the question of whether CRAs can exert an effect on FD in glaucoma patients, with potential implications for structural and functional vision loss.

The primary aim of this study was to investigate possible differences in FD in glaucoma patients with and without CRAs using OCTA scans. The secondary objective was to examine the correlation between the presence of CRA and FD in relation to glaucoma severity.

## 2. Materials and Methods

This prospective, monocentric study was performed in accordance with the ethical standards issued by the ethics committee of the Medical Association of Westfalen-Lippe and the University of Münster; it also adhered to the tenets of the Declaration of Helsinki.

The study was conducted at the Department of Ophthalmology at the University of Muenster Medical Center, Germany. Patient recruitment occurred from February 2017 to August 2022.

Patients diagnosed with primary open-angle glaucoma (POAG), pseudoexfoliation (PEX) glaucoma, pigment-dispersion glaucoma, and normal-tension glaucoma were included in the study. The exclusion criteria included reasons other than glaucoma that might cause abnormalities of the optic nerve head (e.g., optic disc drusen, neovascularization of the optic nerve head, tilted discs, and vascular occlusions). If ophthalmic examination was insufficient to establish such a connection, further testing including neurologic examinations and MRI imaging was performed. Patients showing central retinal pathologies that might influence macular OCTA measurement results were also excluded.

The participants of the study underwent a standardized ophthalmic examination including a refractive eye exam, the examination of the anterior segment, funduscopy, Goldmann applanation tonometry, gonioscopy, perimetry using the automated Humphrey visual field analyzer (HFA II, model 750, Carl Zeiss Meditec AG, Jena, Germany) with the standard program of the 30–2 Swedish interactive threshold algorithm (SITA fast), fundus imaging of the optic disc (VISUCAM, Zeiss, Germany), and OCTA measurements using the RTVue XR Avanti system (AngioVue/RTVue-XR Avanti spectral domain optical coherence tomograph, Optovue Inc., Fremont, CA, USA). Angiographic imaging of the macula used 3 × 3 mm scans, while measurements of the optic disc used 4.5 × 4.5 mm scans. FD, reflecting the ratio (%) of bright to dark pixels (i.e., the ratio of perfused to non-perfused areas) was automatically calculated using the AngioVue algorithm for different retinal layers and sublocations. Twelve parameters, each related to the macular superficial capillary plexus (SCP) and the macular deep capillary plexus (DCP), and 18 parameters related to the radial peripapillary capillaries (RPC) of the optic nerve head were extracted and analyzed. OCTA measurements were performed in a darkened windowless room under the same mesopic lightning conditions by a qualified examiner. Scans with a quality index (QI) <7 were excluded.

The Zeiss VISUCAM photographs and OCTA scans of all individuals included in the study were used to identify CRAs; this was performed by two expert examiners independently. CRAs were characterized as hooked retinal arteries at the rim of the optic nerve head without a connection with the central retinal artery. The eyes were subsequently classified into two cohorts: one cohort included the eyes of glaucoma patients without a CRA, while the other consisted of eyes with at least one CRA. For the severity-adjusted analysis, the CRA and non-CRA patients were further divided into three severity groups according to the perimetry-based level of glaucoma, as suggested by Hodapp et al. [20]. Group 1 (early glaucomatous damage) included patients with a mean deviation (MD) ≥−6 decibels in perimetric testing, Group 2 (moderate glaucomatous damage) included patients with an MD ranging from <−6 to ≥−12 decibels, and Group 3 (advanced glaucomatous damage) included patients with an MD <−12 decibels [20]. Allegedly the type of glaucoma most susceptible to ocular perfusion, we further conducted a separate subanalysis for patients suffering from normal-tension glaucoma (NTG).

The statistical analysis was performed using R, Version 4.1.2. Variables related to the basic characteristics of the patients and eyes included in the study were presented as the absolute and relative frequencies for the categorial variables and as the mean ± standard deviation for continuous variables. The patients with one or more CRAs were grouped together and not analyzed with regard to their exact number of CRAs.

As the normal distribution assumption does not apply to FD variables, we reported the medians of these values; furthermore, we applied non-parametric statistical methods. For comparisons between different groups, we used tests for clustered data as proposed by Rosner et al. as well as their implementation of the R package *clusrank*, and have reported the resulting *p*-values [21,22]. As the clustered data structure should be taken into account when assessing the correlations, rank correlations were applied as suggested by Rosner et al. [21,22,23]. In addition to the point estimates, we also reported the associated 95% confidence intervals.

All analyses were explorative and should be interpreted accordingly. Furthermore, *p*-values below 0.05 and 95% confidence intervals that did not contain 0 were considered noticeable.

## 3. Results

In this study, 201 eyes of 134 primary and secondary open-angle glaucoma patients were included. The patient population characteristics are summarized in Table 1. The majority of the eyes had no CRA (*n* = 145). Among the eyes with CRA (*n* = 56), 44 had one CRA (78.6%), 11 had two CRAs (19.6%), and one had four CRAs (1.8%). Further cohort-specific characteristics are summarized in Table 2.

### 3.1. Comparability of Study Cohorts

Neither age nor the gender ratio differed significantly between the CRA and non-CRA cohorts. Furthermore, neither the imaging-related parameters (such as the mean QI for macular and optic disc images) nor the ocular parameters (such as visual acuity, MD and disease severity, PSD, distribution of glaucoma types, and IOP) were statistically different between the two groups (Table 2).

### 3.2. CRA Versus Non-CRA

Statistical testing revealed noticeably higher FD in the CRA eyes compared to the non-CRA eyes at the level of the DCP. No significant difference was found between the two groups at the level of the SCP or the RPC (Table 3).

As mentioned previously, the CRA and non-CRA eyes were further subdivided according to the Hodapp–Parrish–Anderson classification. These allocations were performed to adjust for glaucoma severity, as this was considered as a possible confounder. The results for the FD comparison between the CRA and non-CRA patients, adjusted for disease severity, are summarized in Table 4. No noticeable differences in FD were observed between the two groups when adjusted for glaucoma severity.

**Figure 2:**
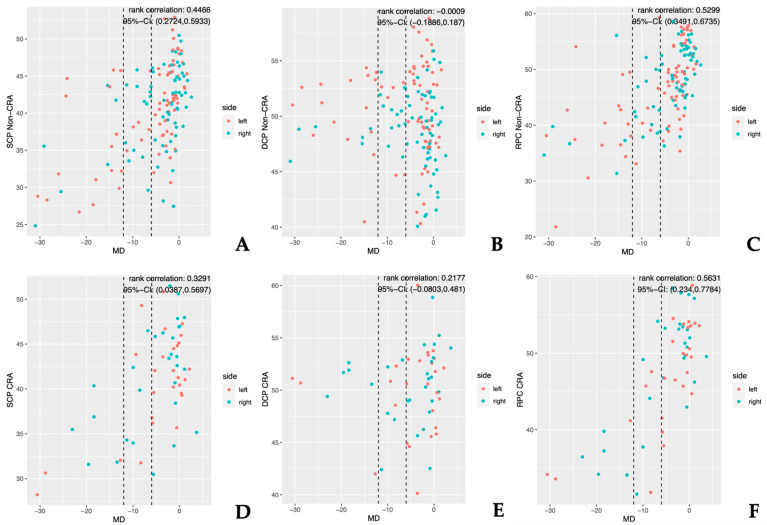
The results of the correlation analysis between the FD values (y-axis) and MD (x-axis) for the entire study population. Glaucoma severity was defined by the MD values. Groups 1 (MD ≥ −6), 2 (MD < −6 ≥ −12), and 3 (MD < −12) are distinguished by dotted lines. Three sectors, which are representative of the results in their respective locations, are each displayed for the CRA and non-CRA eyes. (**A**–**C**) Correlation between the superficial (**A**), deep (**B**), and peripapillary (**C**) FD and MD for the non-CRA eyes; (**D**–**F**) correlation between the superficial (**D**), deep (**E**), and peripapillary (**F**) FD and MD for the CRA eyes. MD = mean deviation; SCP = superficial capillary plexus; DCP = deep capillary plexus; RPC = radial peripapillary capillaries; CRA = cilioretinal artery.

The correlation between the FD reduction and the severity of glaucoma across the CRA and non-CRA eyes was strongest for the RPC sectors, followed by the SCP sectors, while the DCP sectors correlated poorly with the glaucoma severity (Table 5, Figure 2). This was the case even when differentiating between the CRA and non-CRA eyes (Table 5).

Figure 2 illustrates the correlation between FD reduction and disease severity for all the en-face images of the SCP, DCP, and RPC regions for the CRA and non-CRA eyes. Both Figure 2 and Table 5 illustrate that for all parameters, the RPC sectors correlated most strongly with disease severity.

### 3.3. Normal-Tension Glaucoma

The isolated analysis of the eyes of the NTG-patients (*n* = 26) showed noticeably higher FD in the non-CRA eyes (*n* = 17) compared to the CRA eyes (*n* = 9) at the level of the SCP in the total cohort comparison and when adjusted for disease severity (*p* ≤ 0.02). Due to the composition of the NTG cohort, a further disease severity-dependent subanalysis could only reasonably be conducted for early glaucoma patients.

## 4. Discussion

The role of CRAs in glaucoma has always been of considerable interest to researchers. Different authors have evaluated whether CRAs have a protective function with regard to glaucomatous damage. In recent years, this issue has received greater attention, as recent publications have described a negative correlation between FD and the severity of glaucoma. This raises the question of whether an additional vessel could improve FD and, consequently, potentially delay glaucomatous damage. This was the first study to investigate the relationship between FD measured using OCTA and the presence of CRAs in primary and secondary open-angle glaucoma patients.

In a systematic review, the prevalence of CRAs was found to be 6.9–49.5%. [24,25]. In our study, 56 of 201 (27.9%) eyes exhibited at least one CRA. As described in the literature, the vast majority of CRAs in our patients were found temporally (92.9%) [25,26]. The wide range of variation can best be explained by the method of determining the presence of the vessel. Prior to the introduction of OCTA, CRAs could only be identified through funduscopic examination, fundus photography, or fluorescein angiography [24]. We considered the introduction of OCTA helpful in the identification of the vessel (Figure 1) [27].

Both the central retinal artery and the posterior ciliary arteries originate from the ophthalmic artery. The CRAs, in turn, arise from the short posterior ciliary arteries or, in rare instances, directly from the choroidal circulation. As true end arteries, CRAs supply a distinct region of the retina and, in fewer cases, the prelaminar region [28].

Diener et al., analyzed the OCTA-derived FD in healthy patients with and without CRAs. The study group revealed increased FD in the RPC network and in the SCP in patients with CRAs [18]. Notably, we did not observe such changes in our patient cohort. The FD of the CRA and non-CRA patients was comparable in these regions. However, the FD was significantly higher in the CRA patients in the DCP area compared to those without CRA. These differences could arise from the differences in patient populations as vascular regulatory mechanisms may influence the FD measurements more prominently in healthy individuals than in patients with glaucomatous damage to the retina. Vascular theories and vascular regulatory mechanisms have been widely discussed in the literature [29,30,31,32,33].

Alterations in capillary perfusion related to glaucoma have been extensively described in the literature [10,11,12]. Particularly when considering NTG patients, ocular perfusion has been given a special place in the genesis of glaucomatous damage because patients are exposed to relatively low intraocular pressure. Interestingly, in contrast to the overall cohort, when NTG patients were analyzed singularly, a noticeably higher FD was found in the non-CRA patients compared to the CRA patients at the level of SCP.

Even if this result does not allow for any far-reaching conclusions on the possible influence of the presence of a CRA on glaucoma, it can at least be speculated that the presence of an additional blood vessel influences the FD in NTG patients to a higher extent than in the other presented types of glaucoma for the reasons above-mentioned.

Studies investigating POAG patients using OCTA have shown reduced FD around the optic nerve head compared to healthy controls. Furthermore, a correlation was identified between an increase in glaucomatous damage and a decrease in FD [13,34].

This is in line with our results. In our study, FD was negatively correlated with disease severity defined by MD. However, the degree of correlation differed between the macular and papillary regions. The RPC sectors were affected most severely by glaucomatous damage, followed by the SCP sectors and the DCP sectors. Consistent with our findings, Rao et al. reported that the FD of the RPC region could be used more reliably than macular FD to distinguish between healthy eyes and glaucomatous eyes [31]. Taking this into account, the results of our subgroup analysis are of particular interest. While the differences between the groups were not statistically noticeable, a trend toward higher FD in the CRA eyes with early or moderate severity could be identified for the SCP and the RPC compared to the non-CRA eyes. In contrast, for severely affected eyes, FD was lower in the CRA eyes than in the non-CRA eyes in these regions. These observations suggest a shift in FD properties between early/moderate and severely affected glaucomatous eyes. While we cannot provide a definitive answer as to the cause of this shift, this variation between the two cohorts may be explained by a difference in retinal autoregulation, which could behave in a more dysfunctional manner in the CRA eyes than in non-CRA eyes with increasing disease severity. To the best of our knowledge, this is the first study to report such severity-dependent changes in ocular microperfusion.

Nevertheless, the effect of a CRA on the cup–disc ratio, IOP, and visual field defects has been investigated. The groups investigating this issue have found different results: no effect, a protective influence, or a harmful influence on glaucomatous damage have all been attributed to the presence of a CRA in glaucoma patients.

In 2003, Budde et al. examined the influence of the presence and location of CRAs on rim loss and the progression of parapapillary atrophy in glaucoma patients after previous studies had suggested a correlation between the position of the central retinal vessel trunk and glaucomatous damage of the neuroretinal rim; no significant differences were found in the neuroretinal rim area between the CRA and non-CRA groups [28]. Furthermore, two previous studies suggested a protective effect of the vicinity of the central retinal vessel trunk on the lamina cribrosa and on the neighboring segments of the optic disc: optic nerve damage was found to be less extensive in the quadrant containing the central retinal vessel trunk. The authors considered the vessel trunk to be a stabilizing element protecting the optic nerve head from mechanical damage [35,36].

In addition to the hypothesis that a blood vessel may reduce optic nerve damage via mechanical stabilization, another approach concerns the possible alteration of the blood supply to the optic nerve by a CRA.

In their 1988 study, Lindenmuth et al. investigated the relationship between CRAs and glaucomatous optic nerve changes. The study involved 122 individuals with POAG. No significant differences were found between the groups with and without a CRA regarding IOP, the cup–disc ratio, or visual field defects [37].

In their analysis of 20 patients with POAG and unilateral CRAs, Shihab et al. showed a larger cup–disc ratio and more extensive visual field damage in patients with CRAs than in the group without CRAs. The authors assumed the reason for these observations to be the CRA’s ability to shunt blood away from the optic disc, leading to reduced perfusion. They speculated that while CRAs have no negative effect on the eyes of healthy patients, they could cause an increase in glaucomatous damage on the predamaged optic discs [25].

In contrast, when Lee and Schwarzt compared two groups comprising a total of 34 glaucoma patients with and without CRAs, they found that patients with a CRA retained visual field and visual acuity more often. According to the authors, the role of a CRA may be to provide greater circulation to the temporal rim of the optic disc [38].

Due to the small number of eyes included in these studies, Type I and Type II errors must be taken into account when evaluating the respective results. Thus, effects that actually exist may be overlooked or falsely assumed [25,37].

In our study, there were no noticeable differences in the FD between the CRA and non-CRA groups across all glaucoma types when adjusted for glaucoma severity. Subgroup analysis of the NTG patients showed evidence of a noticeable difference in FD at the SCP level between the two groups. We therefore suggest that the influence of CRAs on retinal perfusion in glaucoma patients in general is rather limited, but may be more important in NTG patients.

## 5. Limitations

In our study, the age, the gender ratio, IOP, MD and disease severity, distribution of glaucoma types, mean spherical equivalent, and QI did not differ noticeably between the CRA and non-CRA groups, making it improbable that these factors could have exerted a noticeable confounding effect on the OCTA imaging results. However, as OCTA represents a rather novel imaging technology, our study design might not have accounted for other as-yet-unidentified influencing factors. Furthermore, the spectral-domain technology used in this trial limited the possibility of the high-resolution angiography imaging of retinal layers other than the ones elaborated on. An investigation of choroidal FD using swept-source devices is of interest as both CRAs and choroidal arteries originate from the posterior ciliary arteries. Further longitudinal studies are needed in this regard.

## 6. Conclusions

In conclusion, this investigation showed no noticeable difference in the FD between the CRA and non-CRA glaucomatous eyes when adjusted for glaucoma severity, suggesting that the presence of CRAs does not significantly affect retinal perfusion in glaucoma patients. In both the CRA and non-CRA eyes, increasing glaucoma severity correlated most strongly with a reduction in peripapillary FD. The results support the theory of a disturbed vascular regulatory mechanism in glaucoma patients with regard to the severity of glaucoma. OCTA may help in developing a better understanding of how blood supply might affect glaucomatous damage.

## Figures and Tables

**Table 1:** General patient characteristics. Data on continuous variables are reported as the mean (± standard deviation) or the median (25th percentile; 75th percentile), depending on the data distribution.

Eyes (*n*)	201
Patients (*n*)	134
Age (years) *	62.01 ± 15.82
Gender (M:F)	57:77
Study eye (R:L)	98:103
Eyes (n) according to type of glaucoma	
Primary open angle glaucoma	132 (65.7%)
Pseudoexfoliation glaucoma	33 (16.4%)
Normal-tension glaucoma	26 (12.9%)
Pigment dispersion glaucoma	10 (5.0%)
Visual acuity (logMAR) **	0.1 (0; 0.2)

*n* = number; M = male; F = female; R = right; L = left; logMAR = logarithm of minimum angle of resolution; * (mean ± standard deviation); ** (median (25th percentile; 75th percentile)).

**Table 2:** Patient characteristics according to the CRA group. Data on the continuous variables are reported as the mean (± standard deviation) or the median (25th percentile; 75th percentile), depending on the data distribution.

	non-CRA	CRA	*p*-Value
eyes (*n*)	145	56	
Location of CRA			
Temporal (%)		52 (92.9%)	
Nasal (%)		4 (7.1%)	
Age (years) *	66.8 (58.0; 74.1)	62.8 (55.5; 69.5)	0.08
Gender (M:F)	42:63	22:27	0.83
Study eye (R:L)	69:76	29:27	0.57
Eyes (n) according to type of glaucoma			0.29
Primary open angle glaucoma	93 (64.1%)	39 (70.0%)	
Pseudoexfoliation glaucoma	28 (19.3%)	5 (8.9%)	
Normal-tension glaucoma	17 (11.7%)	9 (16.1%)	
Pigment dispersion glaucoma	7 (4.8%)	3 (5.4%)	
IOP (mmHg) *	14 (12; 17)	14 (12; 16.5)	0.68
MD (dB) *	−2.43 (−6.68; −0.74)	−2.06 (−8.25; −0.32)	0.85
PSD *	2.81 (1.85; 6.74)	2.45 (1.65; 9.40)	0.72
Visual acuity (logMAR)*	0.10 (0.20; 0.00)	0.10 (0.18; 0.10)	0.91
Quality index *			
– macular images	8 (7; 8)	8 (7; 8)	0.15
– papillary images	8 (7; 8)	8 (7; 8)	0.76
Eyes (n) according to disease severity			0.99
(Hodapp-Parrish-Anderson Classification)			
Group 1 (early glaucoma)	104 (71.7%)	40 (71.4%)	
Group 2 (moderate glaucoma)	19 (13.1%)	8 (14.3%)	
Group 3 (advanced glaucoma)	22 (15.2%)	8 (14.3%)	

*n* = number; M = male; F = female; R = right; L = left; CRA = cilioretinal artery; IOP = intraocular pressure; mmHg = millimeters of mercury; MD = mean deviation; dB = decibel; PSD = pattern standard deviation; logMAR = logarithm of minimum angle of resolution; * (median (25th percentile; 75th percentile)).

**Table 3:** FD in the CRA and non-CRA groups measured by OCTA.

	Location	Non-CRA	CRA	*p*-Value
SCP	Whole en face	41.73	42.04	0.81
	Whole en face superior hemisphere	41.55	41.54	0.96
	Whole en face inferior hemisphere	41.34	42.18	0.71
	ETDRS	40.94	41.89	0.80
	Fovea	18.24	19.73	0.26
	Para Fovea	43.84	44.47	0.94
	Para Fovea superior hemisphere	43.66	44.07	0.82
	Para Fovea inferior hemisphere	43.79	44.93	0.70
	Para Fovea temporal	42.29	42.56	0.78
	Para Fovea superior	44.7	44.95	0.99
	Para Fovea nasal	42.80	44.36	0.27
	Para Fovea inferior	45.66	45.73	0.63
DCP	Whole en face	49.6	50.77	0.39
	Whole en face superior hemisphere	49.25	51.36	0.07
	Whole en face inferior hemisphere	48.87	50.52	0.11
	ETDRS	48.58	50.74	**0.05**
	Fovea	33.11	33.31	0.33
	Para Fovea	51.68	52.91	0.42
	Para Fovea superior hemisphere	50.91	53.63	**0.03**
	Para Fovea inferior hemisphere	50.88	52.78	0.10
	Para Fovea temporal	50.49	52.06	0.23
	Para Fovea superior	51.11	53.81	**0.03**
	Para Fovea nasal	50.96	53.88	**0.03**
	Para Fovea inferior	50.77	53.8	**0.05**
RPC	Whole en face	48.34	49.54	0.79
	Whole en face capillaries	42.19	43.77	0.89
	Inside disc all	54.93	55.05	0.96
	Inside disc capillaries	47.20	47.04	0.93
	Peripapillary all	50.85	51.26	0.60
	Peripapillary capillaries	44.85	45.68	0.94
	Superior hemisphere all	51.74	52.17	0.91
	Inferior hemisphere all	49.91	51.9	0.72
	Superior hemisphere capillaries	45.65	46.86	0.84
	Inferior hemisphere capillaries	43.79	46.25	0.92
	Nasal superior	42.92	44.07	0.77
	Nasal inferior	40.29	41.59	0.59
	Inferior nasal	42.53	43.60	0.93
	Inferior temporal	50.61	54.59	0.80
	Temporal inferior	47.63	48.76	0.59
	Temporal superior	51.6	53.08	0.37
	Superior temporal	48.00	46.52	0.26
	Superior nasal	41.52	41.93	0.63

SCP = superficial capillary plexus; DCP = deep capillary plexus; RPC = radial peripapillary capillaries; CRA = cilioretinal artery; parafovea = area surrounding the fovea; ETDRS = Early Treatment Diabetic Retinopathy Study grid; All *p*-values < 0.05 are emboldened.

**Table 4:** Difference in FD between the CRA and non-CRA patients, adjusted for glaucoma severity.

		Group 1 MD: ≥ −6 dB (Early)			Group 2 MD: −6–≥ −12 dB (Moderate)			Group 3 MD: < −12 dB (Advanced)		
	Location	Non-CRA	CRA	*p*-Value	Non-CRA	CRA	*p*-Value	Non-CRA	CRA	*p*-Value
SCP	Whole en face	42.46	43.01	0.57	38.75	41.13	0.74	32.68	31.95	0.76
	Whole en face superior hemisphere	42.16	42.64	0.60	41.2	42.33	0.76	34.15	34.38	0.67
	Whole en face inferior hemisphere	42.34	43.53	0.42	40.92	44.11	0.38	30.32	31.16	0.84
	ETDRS	41.82	42.54	0.49	39.26	42.44	0.38	32.36	31.67	0.92
	Fovea	19.38	20.8	0.39	14.49	18.27	0.47	16.00	15.9	0.87
	Para Fovea	45.07	45.73	0.77	42.08	44.09	0.67	35.11	33.79	0.91
	Para Fovea superior hemisphere	44.11	44.84	0.37	43.1	43.3	0.49	36.66	36.14	0.76
	Para Fovea inferior hemisphere	44.92	46.02	0.46	43.18	48.35	0.28	31.55	33.23	0.68
	Para Fovea temporal	43.38	43.74	0.90	39.15	43.92	0.38	31.86	33.7	0.90
	Para Fovea superior	44.96	45.88	0.61	45.06	44.18	0.91	37.66	36.15	0.92
	Para Fovea nasal	43.39	45.86	0.16	43.71	44.83	0.45	36.89	36.30	0.76
	Para Fovea inferior	46.33	47.63	0.94	44.71	50.83	0.28	32.32	35.08	0.29
DCP	Whole en face	49.55	50.85	0.31	50.58	49.71	0.81	49.26	50.9	0.54
	Whole en face superior hemisphere	48.25	51.64	0.13	50.99	50.83	0.68	49.25	51.34	0.17
	Whole en face inferior hemisphere	48.81	50.46	0.19	49.16	50.87	0.82	48.71	50.52	0.20
	ETDRS	48.34	50.84	0.10	49.82	49.66	0.82	48.58	50.69	0.18
	Fovea	34.22	35.97	0.18	31.96	29.35	0.64	30.22	30.43	0.94
	Para Fovea	51.36	52.49	0.33	53.34	51.44	0.55	51.63	53.35	0.48
	Para Fovea superior hemisphere	50.25	53.6	0.07	53.17	52.17	0.82	51.45	53.74	0.09
	Para Fovea inferior hemisphere	50.55	52.32	0.13	52.24	53.99	0.66	50.85	53.18	0.46
	Para Fovea temporal	50.38	52.5	0.23	52.08	53.41	0.87	50.43	51.50	0.79
	Para Fovea superior	50.84	53.18	0.07	53.01	51.35	0.82	51.71	54.43	0.10
	Para Fovea nasal	50.42	53.67	0.08	52.51	54.44	0.62	52.07	53.44	0.39
	Para Fovea inferior	50.57	52.98	0.14	51.71	53.86	0.58	52.47	54.32	0.06
RPC	Whole en face	49.81	51.43	0.43	42.71	44.9	0.80	39.10	35.32	0.24
	Whole en face capillaries	43.94	45.33	0.66	38.22	40.49	0.45	29.34	28.74	0.39
	Inside disc all	56.9	57.04	0.69	53.8	56.03	0.76	49.87	49.94	0.99
	Inside disc capillaries	49.02	49.05	0.99	46.41	47.06	0.66	42.31	44.73	0.67
	Peripapillary all	52.25	54.14	0.23	43.9	46.51	0.75	37.45	34.37	0.36
	Peripapillary capillaries	46.81	48.57	0.41	39.62	42.29	0.72	28.38	27.69	0.28
	Superior hemisphere all	52.96	54.32	0.51	45.41	46.1	0.66	38.48	37.57	0.52
	Inferior hemisphere all	52.92	53.44	0.20	45.61	52.55	0.49	36.02	32.51	0.28
	Superior hemisphere capillaries	47.72	48.65	0.69	38.45	38.52	0.66	29.69	29.66	0.99
	Inferior hemisphere capillaries	47.19	47.45	0.35	38.27	46.57	0.49	27.13	24.83	0.27
	Nasal superior	44.40	45.65	0.25	40.95	37.08	0.66	27.42	25.89	0.83
	Nasal inferior	42.21	43.68	0.26	37.87	46.98	0.62	30.34	28.80	0.79
	Inferior nasal	45.37	45.68	0.55	36.26	41.17	0.49	21.47	18.13	0.63
	Inferior temporal	52.63	55.21	0.14	36.47	55.63	0.62	25.24	18.79	0.25
	Temporal inferior	48.92	51.92	0.14	48.71	46.74	0.98	35.81	32.76	0.38
	Temporal superior	52.11	53.76	0.40	53.02	55.96	0.25	40.51	42.68	0.60
	Superior temporal	50.76	50.79	0.77	39.35	29.49	0.31	25.27	25.96	0.67
	Superior nasal	44.04	44.33	0.91	35.87	27.67	0.37	25.93	23.37	0.47

MD = mean deviation; dB = decibel; SCP = superficial capillary plexus; DCP = deep capillary plexus; RPC = radial peripapillary capillaries; CRA = cilioretinal artery; parafovea = area surrounding the fovea; ETDRS = Early Treatment Diabetic Retinopathy Study grid.

**Table 5:** Correlation analysis between the FD reduction and the severity of glaucoma for the CRA and non-CRA cohorts.

		CRA			Non-CRA		
	Location	Estimate	Lower 95%-CI Bound	Upper 95%-CI Bound	Estimate	Lower 95%-CI Bound	Upper 95%-CI Bound
SCP	Whole en face	0.33	0.04	0.57	0.45	0.27	0.59
	Whole en face superior hemisphere	0.40	0.01	0.69	0.47	0.24	0.65
	Whole en face inferior hemisphere	0.30	−0.16	0.65	0.56	0.36	0.72
	ETDRS	0.30	−0.16	0.65	0.54	0.33	0.69
	Fovea	0.16	−0.16	0.45	0.26	0.08	0.42
	Para Fovea	0.34	0.02	0.60	0.43	0.26	0.58
	Para Fovea superior hemisphere	0.26	−0.35	0.72	0.46	0.21	0.65
	Para Fovea inferior hemisphere	0.34	−0.07	0.66	0.52	0.31	0.69
	Para Fovea temporal	0.35	−0.20	0.74	0.52	0.30	0.68
	Para Fovea superior	0.22	−0.21	0.59	0.43	−0.93	0.99
	Para Fovea nasal	0.25	−0.16	0.58	0.43	0.26	0.55
	Para Fovea inferior	0.32	0.01	0.57	0.54	0.33	0.69
DCP	Whole en face	0.22	−0.08	0.48	0.00	−0.19	0.19
	Whole en face superior hemisphere	0.25	−0.32	0.69	−0.11	−0.48	0.3
	Whole en face inferior hemisphere	0.36	−0.14	0.72	0.05	−0.37	0.46
	ETDRS	0.29	−0.35	0.75	0.00	−0.45	0.44
	Fovea	0.23	−0.18	0.57	0.25	0.07	0.41
	Para Fovea	0.16	−0.18	0.47	−0.03	−0.23	0.16
	Para Fovea superior hemisphere	0.25	−0.66	0.87	−0.14	−0.50	0.26
	Para Fovea inferior hemisphere	0.26	−0.35	0.71	−0.02	−0.40	0.36
	Para Fovea temporal	0.32	−0.35	0.78	0.02	−0.51	0.53
	Para Fovea superior	0.10	−0.98	0.99	−0.20	−0.59	0.26
	Para Fovea nasal	0.28	−0.15	0.62	−0.06	−0.36	0.26
	Para Fovea inferior	0.13	−0.25	0.47	−0.05	−0.37	0.29
RPC	Whole en face	0.56	0.23	0.78	0.53	0.35	0.67
	Whole en face capillaries	0.57	−1.00	1.00	0.57	0.36	0.73
	Inside disc all	0.38	−0.46	0.86	0.31	0.03	0.55
	Inside disc capillaries	0.27	−0.82	0.94	0.27	−0.05	0.54
	Peripapillary all	0.61	0.20	0.84	0.53	0.35	0.67
	Peripapillary capillaries	0.59	0.43	0.72	0.59	0.37	0.75
	Superior hemisphere all	0.52	−0.75	0.97	0.53	0.30	0.70
	Inferior hemisphere all	0.46	−0.03	0.77	0.55	0.33	0.72
	Superior hemisphere capillaries	0.53	−0.06	0.85	0.54	0.31	0.71
	Inferior hemisphere capillaries	0.53	−0.28	0.90	0.56	0.33	0.72
	Nasal superior	0.49	−0.03	0.80	0.55	0.32	0.72
	Nasal inferior	0.28	−0.12	0.61	0.42	0.25	0.54
	Inferior nasal	0.48	−0.07	0.81	0.48	0.25	0.66
	Inferior temporal	0.62	0.07	0.88	0.56	0.30	0.74
	Temporal inferior	0.41	−0.26	0.82	0.46	−0.99	1.00
	Temporal superior	0.07	−1.00	1.00	0.40	0.12	0.62
	Superior temporal	0.65	−0.66	0.98	0.46	0.16	0.69
	Superior nasal	0.55	0.14	0.81	0.46	0.12	0.70

SCP = superficial capillary plexus; DCP = deep capillary plexus; RPC = radial peripapillary capillaries; CRA = cilioretinal artery; parafovea = area surrounding the fovea; ETDRS = Early Treatment Diabetic Retinopathy Study grid; Note that positive estimates translate into a reduction in FD for an increase in disease severity.

## Data Availability

Not applicable.
